# COVID‐19 vaccine hesitancy: The synergistic effect of anxiety and proactive coping

**DOI:** 10.1002/puh2.70

**Published:** 2023-03-05

**Authors:** MacKenzie L. Hughes, Shevaun D. Neupert, Emily L. Smith, Clara W. Coblenz, Samuel G. Macy, Ann Pearman

**Affiliations:** ^1^ School of Psychology Georgia Institute of Technology Atlanta Georgia USA; ^2^ Department of Psychology North Carolina State University Raleigh North Carolina USA; ^3^ MetroHealth Medical Center Cleveland Ohio USA

**Keywords:** anxiety, COVID‐19, pandemic, proactive coping, vaccination attitudes, vaccine hesitancy

## Abstract

**Background:**

This study sought to identify cognitive and behavioral predictors of COVID‐19 vaccine hesitancy. Specifically, this study examined the effect of anxiety about developing COVID‐19 and proactive coping behavior on the likelihood of reporting COVID‐19 vaccine hesitancy in a sample of adults living in the United States.

**Methods:**

An online survey of proactive coping strategies, anxiety related to developing COVID‐19, and vaccine hesitancy was administered in October 2020 to 534 adults aged 21–79‐years old. Age, gender, race, self‐rated health, years of education, COVID‐19 knowledge, and perceived constraints were included as covariates.

**Results:**

Over half of the study participants (56.7%) were COVID‐19 vaccine hesitant. People who were less anxious about developing COVID‐19 were more likely to be vaccine hesitant. A statistically significant COVID‐19 anxiety × proactive coping interaction showed the odds of vaccine hesitancy was highest among individuals with low anxiety about developing COVID‐19 and high proactive coping, whereas vaccine hesitancy was lowest among individuals with high COVID‐19 anxiety and high proactive coping.

**Conclusion:**

Results support a future‐oriented approach to public health outreach efforts regarding COVID‐19 vaccines. Improvement of proactive coping skills and emphasis on the likelihood of contracting COVID‐19 may be more effective in increasing vaccine uptake than simply restating scientific facts regarding safety or efficacy.

To reduce the spread of COVID‐19, high rates of vaccine uptake are required globally [1]. However, building population level trust around vaccine safety and functionality is complex and highly driven by bio and psychosocial factors [2]. Vaccine hesitancy has continued to challenge efforts toward achieving herd immunity [3]. Despite efforts to address vaccine skepticism and mistrust by increasing public health messaging focused on vaccine safety, COVID‐19 vaccination rates remain suboptimal in the United States [4]. Repeating scientific evidence to debunk vaccine misinformation and demonstrate vaccine safety and effectiveness may not be enough to change anti‐vaccination attitudes and beliefs [5]. Identifying predictors of vaccine hesitancy may provide alternative avenues for tailoring public health messaging to increase vaccination uptake and encourage people to continue to stay up to date on their vaccines.

This study explored the effect of proactive coping and anxiety about developing COVID‐19 on vaccine hesitancy. Proactive coping and anxiety about contracting COVID‐19 reflect future‐oriented behaviors and cognitions, which may have a role in the adoption of health‐related behavior. Recent research showed that people with a future‐oriented thinking style were more likely to take the COVID‐19 vaccine and engage in precautionary health‐related behaviors, such as social distancing and wearing a mask [5]. Proactive coping represents effortful steps to modify or avoid a stressor before it occurs [6]. It is associated with the adoption of behaviors known to delay or prevent undesirable health outcomes [6]. Anxiety specific to developing COVID‐19 is associated with higher levels of stress, especially for older adults [7]. Although anxiety is typically associated with poor health outcomes [8], anxiety in response to risks associated with an illness is an affective component of risk perception [9]. That is, anxiety may be an important cognitive underpinning tied to vaccine uptake because anxiety reflects one's belief in the dangerousness of the virus and risk of contracting it. Previous work showed that COVID‐19‐specific anxiety, but not unspecific anxiety, was negatively associated with COVID‐19 vaccine hesitancy in Germany [8].

This study identified predictors of vaccine hesitancy prior to the release of the vaccine in a sample of adults living in the United States. Given previous research showing a negative relationship between COVID‐19‐specific anxiety and vaccine hesitancy [8], we anticipated that people with less anxiety about developing COVID‐19 would be more hesitant to take the vaccine. Because proactive coping is associated with the adoption of health‐promoting behaviors [6], we also expected that people who engaged in less proactive coping would be more likely to be vaccine hesitant. However, because there are no published studies examining the effect of proactive coping on COVID‐19 vaccine hesitancy, this study explored the interaction between COVID‐19 anxiety and proactive coping without making hypotheses about the direction of the effects.

## METHODS

### Study design, participants, and setting

This study was part of a larger 21‐day online daily diary study that began data collection in October 2020. The current study focused specifically on data collected in the first survey. We recruited adults across the lifespan to understand their perceptions related to the COVID‐19 vaccine. To be eligible for the study, individuals had to be 21–79‐years old, live in the United States, identify as either White or Black or African American and had to be English speaking. The minimum age of 21 was chosen because the survey from the larger study included questions related to alcohol consumption and the minimum drinking age in the United States is 21 years. We focused specifically on the experiences of White and Black Americans because of the prominence of social movements occurring during data collection, including the Black Lives Matter movement, as well as racial disparities in COVID‐19 vaccination. We wanted to ensure our sample included Black Americans so that we could understand and contrast this racial group's experiences to that of White Americans during this time in the United States.

### Study variables, instrument, and data collection

A number of variables were considered in this study. For “COVID‐19 vaccine hesitancy,” participants responded “agree,” “disagree,” or “I don't know” to the item, “If there is a vaccine developed for COVID‐19, I will definitely get one.” Participants that answered either “disagree” or “I don't know” were categorized as vaccine hesitant. “COVID‐19 anxiety” was measured using a scale from 1 (*not at all anxious*) to 5 (*very anxious*), and participants answered, “How anxious are you about developing COVID‐19?” [7, 10]. “Proactive coping” used the 6‐item proactive coping scale [6] ranging from 1 (*strongly disagree*) to 5 (*strongly agree*). The scale measures individuals’ tendencies to plan ahead. An example item includes, “I prepare for adverse events.” Higher scores indicate more proactive coping (Cronbach's alpha = 0.74).

Participants were recruited using Qualtrics Panels and Amazon Mechanical Turk. The survey was hosted on Qualtrics, an online survey platform. The survey took approximately 30 min to complete.

### Data analysis

Data were analyzed using SAS software version 9.4. Logistic regression was used to identify cognitive and behavioral predictors of vaccine hesitancy (0 = *not hesitant*, 1 = *hesitant*). Age, gender, race, education, COVID‐19 knowledge [7], self‐rated health, and perceived constraints [11] were included as covariates for their association with vaccine hesitancy and relevance to understanding people's experiences during the pandemic [12]. The study and the statistical analysis plan were not preregistered. The data are not publicly available online, but de‐identified data and analytic code will be made available upon request.

### Ethical considerations

The study was approved by the Georgia Institute of Technology Institutional Review Board. Informed consent was obtained electronically. Participants were given $5.00 for their participation.

## RESULTS

The study sample included 534 adults (40.1% male, 59.9% female) aged 21–79‐years old (*M_age_* = 45.40, SD = 15.40). The sample was 53.8% White and 46.2% Black or African American. Participants lived in 42 states across the United States. Table 1 reports descriptive information and intercorrelations for each study variable. Over half of the student participants (56.7%) were COVID‐19 vaccine hesitant.

**TABLE 1:** Descriptive statistics and intercorrelations.

Variables	Mean	SD	1	2	3	4	5	6	7	8	9
1. Vaccine hesitancy	0.57	0.50	–								
2. Age	45.40	15.40	−0.12**	–							
3. Gender	0.60	0.49	*6.49* *	0.14**	–						
4. Race	0.46	0.50	*18.44* ***	−0.43***	*2.71*	–					
5. Education	13.59	4.03	−0.06	0.18***	−0.05	−0.16***	–				
6. Self‐rated health	3.58	1.05	−0.06	−0.14***	−0.14***	0.11*	0.05	–			
7. COVID‐19 knowledge	21.57	6.77	−0.22***	0.36***	0.06	−0.31***	0.28***	−0.04	–		
8. Constraints	3.48	1.54	−0.05	−0.21***	−0.06	−0.01	−0.10*	−0.03	−0.28***	–	
9. COVID‐19 anxiety	2.90	1.51	−0.17***	0.07	0.01	−0.10*	0.15***	−0.19***	0.20***	0.16***	–
10. Proactive coping	3.82	0.73	−0.09*	0.18***	0.01	−0.11*	0.20***	0.15***	0.34***	−0.29***	−0.01

*Note*: Vaccine hesitancy coded 0 = *not vaccine hesitant*, 1 = *vaccine hesitant*. Gender coded 0 = *male*, 1 = *female*. Race coded 0 = *White*, 1 = *Black, or African American*. *χ* ^2^ reported for bivariate correlations, indicated in italics. Vaccine hesitancy was disproportionately female, *χ* ^2^(1) = 6.49, *p* < 0.05. Vaccine hesitancy had a greater number of Black or African Americans, *χ* ^2^(1) = 18.44, *p* < 0.0001. Gender was equally distributed across race, *χ* ^2^(1) = 2.71, *p* = 0.10.

* *p* < 0.05.

^**^p < 0.01.

^***^p < 0.001.

Table 2 reports the results of the logistic regression. Individuals were more likely to be vaccine hesitant if they were women, had less COVID‐19 knowledge, and had lower perceived constraints. There was a significant main effect of COVID‐19 anxiety, such that people with lower anxiety were more likely to be hesitant. Although there was no particular significant effect of proactive coping, there was a significant COVID‐19 anxiety × proactive coping interaction.

**TABLE 2:** Results from logistic regression predicting vaccine hesitancy.

Variables	Estimate	Standard error	Odds ratios	95% confidence intervals
Intercept	−0.24	0.17	0.79	(0.56, 1.10)
Age	−0.01	0.01	0.99	(0.98, 1.01)
Gender	0.59	0.20	1.80*	(1.21, 2.67)
Race	0.42	0.22	1.52	(0.99, 2.34)
Education	0.01	0.03	1.02	(0.97, 1.07)
Self‐rated health	−0.18	0.10	0.84	(0.69, 1.02)
COVID‐19 knowledge	−0.06	0.02	0.94***	(0.90, 0.97)
Constraints	−0.15	0.07	0.86*	(0.75, 0.99)
COVID‐19 anxiety	−0.17	0.07	0.84*	(0.74, 0.97)
Proactive coping	−0.09	0.15	0.91	(0.68, 1.23)
Anxiety × proactive coping	−0.23	0.09	0.79*	(0.66, 0.94)

*Note*: Outcome variable coded 0 = *not vaccine hesitant*, 1 = *vaccine hesitant*. Gender coded 0 = *male*, 1 = *female*. Race coded 0 = *White*, 1 = *Black or African American*. Nagelkerke's *R* ^2^ = 0.16.

* *p* < 0.05.

^**^p < 0.01.

^***^p < 0.001.

**FIGURE 1:**
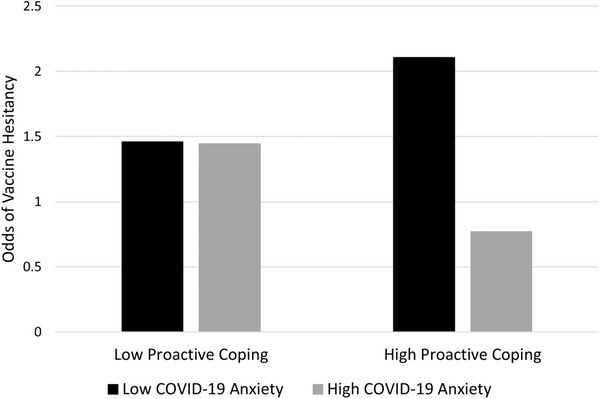
COVID‐19 anxiety × proactive coping interaction. All study covariates were included in the model. Low and high COVID‐19 anxiety were operationalized as 1 SD below and above the mean, respectively. Low and high proactive coping were operationalized as 1 SD below and above the mean, respectively. The resulting raw scores were converted into odds of COVID‐19 vaccine hesitancy.

Figure 1 shows that the odds of vaccine hesitancy were highest among individuals with low anxiety and high proactive coping, whereas hesitancy was lowest among individuals with high anxiety and high proactive coping.

## DISCUSSION

Vaccine hesitancy not only challenges efforts toward achieving herd immunity [2] but also may be a contributing factor to vaccine wastage [13]. The goal of the current study was to examine possible cognitive (represented by anxiety about contracting COVID‐19) and behavioral (measured by proactive coping) underpinnings of COVID‐19 vaccine hesitancy. The study found that people with low levels of COVID‐19 anxiety and high levels of proactive coping were the most likely to be vaccine hesitant. When anxiety specifically related to contracting an illness is low, motivation to prevent the illness may also be low. Bendau et al. showed that COVID‐19‐related anxiety but not unspecific anxiety was positively related to vaccine acceptance [8]. Results suggest that beliefs about personal risk of contracting COVID‐19 combined with coping behavior are important for vaccine uptake. Synergies among risk appraisals (i.e., anxiety about developing COVID‐19) and coping could make for effective behavior change interventions [14].

The relationship between COVID‐19 anxiety and vaccine hesitancy supports the importance of affective risk perceptions related specifically to the development of COVID‐19. Our findings provide evidence that increasing future‐oriented competencies such as proactive coping could be beneficial for vaccine uptake. Proactive coping includes a modifiable set of skills that can be improved through training [15]. In combination with helping individuals understand the risk of developing COVID‐19, encouraging individuals to anticipate both the benefits of taking the vaccine and the potential consequences of not taking the vaccine could provide an alternative avenue for public health messaging to enhance vaccine uptake. In the context of health‐related decision‐making, another beneficial impact of future‐oriented thinking could include the tendency for individuals who engage in proactive coping to update their risk appraisals of developing COVID‐19 by regularly seeking updated information about the development and treatment of the disease [6].

Data were collected before vaccines were widely available, so in addition to the future‐oriented nature of our predictors, participants needed to forecast their vaccine decision. This is a real‐world representation of ongoing vaccine‐related decisions given there are still many unvaccinated adults in the United States [4] and in order to keep vaccinations up to date, the COVID‐19 vaccine regimen may become similar to that of the annual influenza vaccine, which is updated to target the circulating strain.

We acknowledge some limitations of the study. Specifically, the COVID‐19 anxiety measure was a single item, and the data were collected before a COVID‐19 vaccine was widely available and certainly before a vaccine was fully FDA approved. In addition, our results may underestimate the effect of anxiety that could be specific to vaccines because our measure was focused on getting COVID‐19 rather than anxiety specifically related to vaccine uptake or side effects. The notable strengths of the study include the nationally based sample, wide age range, and representation of White and Black participants.

## CONCLUSION

Our results provide a foundation for future research and intervention development focused on the synergistic effect of COVID‐19 anxiety and proactive coping on vaccine hesitancy. Reducing infection rates and COVID‐19‐related mortality depends on vaccine uptake and public adherence to other preventive health behaviors. Without adopting new approaches to overcome high rates of COVID‐19 vaccine hesitancy, public health challenges associated with low vaccination rates will continue, especially given that people will likely need to continually stay up to date with their COVID‐19 vaccine, much like the annual influenza vaccine.

## AUTHOR CONTRIBUTIONS

*Formal analysis; project administration; writing and original draft*: MacKenzie L. Hughes. *Conceptualization; supervision; writing and original draft*: Shevaun D. Neupert. *Conceptualization; writing and original draft*: Emily L. Smith, Clara W. Coblenz, Samuel G. Macy. *Conceptualization; data curation; funding acquisition; investigation; methodology; project administration; supervision; writing and original draft*: Ann Pearman.

## CONFLICT OF INTEREST STATEMENT

The authors declare that they have no conflict of interest.

## ETHICS STATEMENT

All procedures performed in this study were approved by the Georgia Institute of Technology Institutional Review Board.

## Data Availability

These data will be made available upon reasonable request due to IRB restrictions.
